# Posttraumatic* Sphingomonas paucimobilis* Endophthalmitis

**DOI:** 10.1155/2015/192864

**Published:** 2015-12-29

**Authors:** Konstantinos Droutsas, Georgios Kalantzis, Chrysanthos Symeonidis, Ilias Georgalas

**Affiliations:** ^1^1st Department of Ophthalmology, University of Athens, General Hospital “G. Gennimatas”, Mesogeion Avenue 158, 156 69 Athens, Greece; ^2^2nd Department of Ophthalmology, Aristotle University of Thessaloniki, “Papageorgiou” General Hospital, Thessaloniki Ring Road, 564 03 Thessaloniki, Macedonia

## Abstract

A rare case of* Sphingomonas paucimobilis* endophthalmitis secondary to a penetrating globe injury with a retained intraocular foreign body is presented. A 30-year-old man presented with severe pain following a penetrating left eye injury. Visual acuity (VA) was 6/120. Slit-lamp examination revealed perforation of the temporal cornea and iris, hypopyon, and a fibrinous membrane covering the pupil. Ultrasonography showed dense vitreous infiltration and an orbital CT-scan confirmed the presence of a metallic foreign body in the vitreous cavity. Topical and systemic therapy were initiated. Pars-plana vitrectomy combined with phacoemulsification was performed in order to remove the foreign body; vitreous samples were acquired and* Sphingomonas paucimobilis*, sensitive to ceftazidime, was identified. To the best of our knowledge, this is the first report of* Sphingomonas paucimobilis* endophthalmitis following penetrating ocular injury. In this case,* Sphingomonas paucimobilis* was not resistant to antibiotics. This allowed for a good healing response following vitrectomy despite the fact that long-term retinal complications resulted in low VA.

## 1. Introduction

*Sphingomonas paucimobilis* is an aerobic Gram-negative soil bacillus that can be isolated in a variety of environments. A number of* Sphingomonas* species, especially* S. paucimobilis*, may be commonly detected in hospital equipment such as temperature probes, humidifiers, bedside water containers, and sinks.

This is an interventional case report of a culture-proven case of* Sphingomonas paucimobilis* endophthalmitis due to a penetrating globe injury with a retained intraocular foreign body (IOFB).

## 2. Case Report

**Figure 1:**
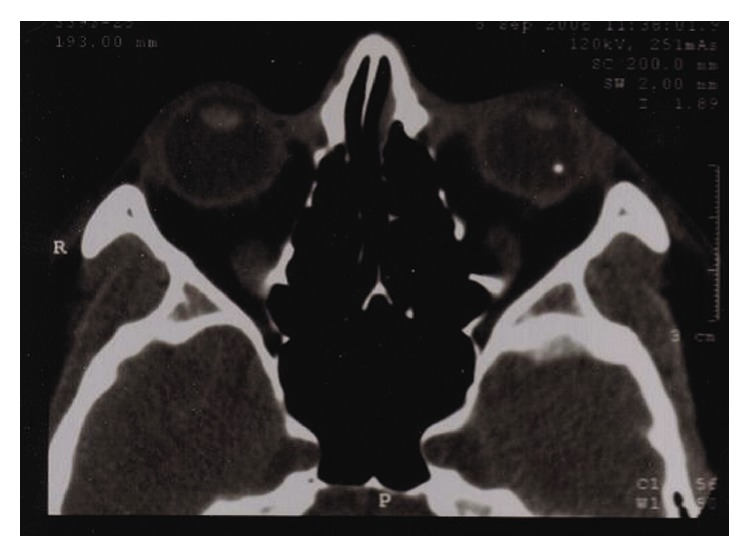
CT-scan depicting an intraocular foreign body in the left eye.

**Figure 2:**
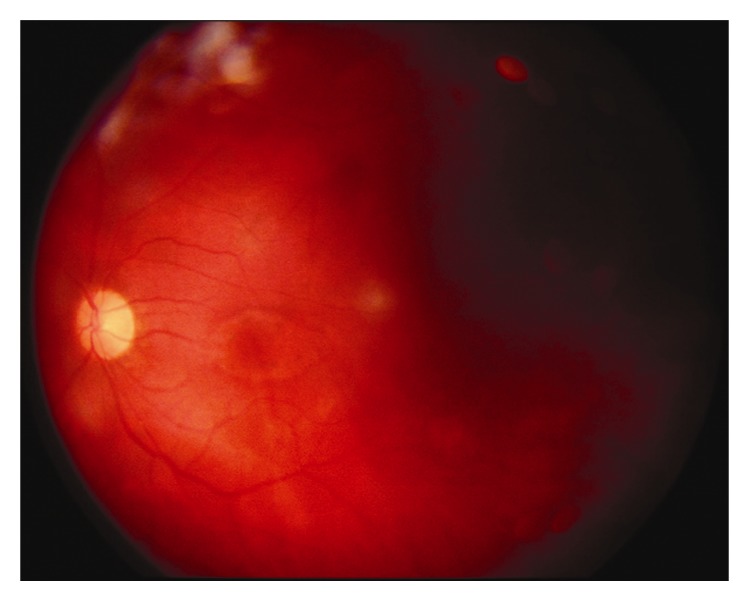
Color fundus picture depicting endophthalmitis.

A 30-year-old-man presented with a visual acuity (VA) of 6/120 and severe ocular pain following a penetrating left eye injury. Slit-lamp examination revealed perforation of the temporal cornea and iris; in the anterior chamber, there was hypopyon and a fibrinous membrane covering the pupil. Ultrasonography showed dense vitreous infiltration while a computerized tomography (CT) orbital scan confirmed the presence of a metallic IOFB in the vitreous cavity (Figure 1). Ophthalmic and systemic treatment were initiated (vancomycin drops and amikacin drops every 2 hours, atropine 1% twice daily, intravenous vancomycin 1 g three times daily, and intravenous amikacin 750 mg twice daily). The IOFB was removed with a pars-plana vitrectomy combined with phacoemulsification and undiluted vitreous specimens were sent for laboratory testing (Figure 2). An oxidase positive, Gram-negative aerobic bacterium with yellow pigmented colonies was isolated.* Sphingomonas paucimobilis* was identified and found to be sensitive to aminoglycosides, tetracycline, chloramphenicol, and ceftazidime. Despite the initially excellent response (VA: 6/9) six months postoperatively, eight months postoperatively, a stage 3 macular hole was observed as well as severe VA deterioration. A second vitrectomy was performed, but VA remained low over the following 24 months despite macular hole closure.

## 3. Discussion

This is the first report of* Sphingomonas paucimobilis* endophthalmitis, a rare ocular infection caused by an environmental bacterium of low virulence, following penetrating ocular injury [1, 2]. Two more reports of* Sphingomonas paucimobilis* endophthalmitis following uneventful phacoemulsification have been reported in the literature. According to the former report, the bacterium was resistant to ceftazidime [3, 4], while, in the latter report, endophthalmitis occurred three months postoperatively. The patient was successfully managed with vitrectomy without an improvement in visual acuity [5].

In our case, in contrast to previous reports,* Sphingomonas paucimobilis* was not resistant to antibiotics. Nevertheless, pars-plana vitrectomy was performed as an immediate management option in order to avoid long-term complications. This allowed for a satisfactory healing response despite the fact that retinal complications eventually led to low postoperative visual recovery.
